# Genome-wide analysis of genes involved in efflux function and regulation within *Escherichia coli* and *Salmonella enterica* serovar Typhimurium

**DOI:** 10.1099/mic.0.001296

**Published:** 2023-02-06

**Authors:** Emma R. Holden, Muhammad Yasir, A. Keith Turner, John Wain, Ian G. Charles, Mark A. Webber

**Affiliations:** ^1^​ Quadram Institute Bioscience, Norwich Research Park, Norwich, Norfolk, NR4 7UQ, UK; ^2^​ Norwich Medical School, University of East Anglia, Norwich Research Park, Norwich, Norfolk, NR4 7TJ, UK

**Keywords:** *acrB*, functional genomics, marA, ramA, soxS, TraDIS, Tn-Seq, tolC

## Abstract

The incidence of multidrug-resistant bacteria is increasing globally, with efflux pumps being a fundamental platform limiting drug access and synergizing with other mechanisms of resistance. Increased expression of efflux pumps is a key feature of most cells that are resistant to multiple antibiotics. Whilst expression of efflux genes can confer benefits, production of complex efflux systems is energetically costly and the expression of efflux is highly regulated, with cells balancing benefits against costs. This study used TraDIS-*Xpress*, a genome-wide transposon mutagenesis technology, to identify genes in *Escherichia coli* and *Salmonella* Typhimurium involved in drug efflux and its regulation. We exposed mutant libraries to the canonical efflux substrate acriflavine in the presence and absence of the efflux inhibitor phenylalanine-arginine β-naphthylamide. Comparisons between conditions identified efflux-specific and drug-specific responses. Known efflux-associated genes were easily identified, including *acrAB*, *tolC*, *marRA*, *ramRA* and *soxRS*, confirming the specificity of the response. Further genes encoding cell envelope maintenance enzymes and products involved with stringent response activation, DNA housekeeping, respiration and glutathione biosynthesis were also identified as affecting efflux activity in both species. This demonstrates the deep relationship between efflux regulation and other cellular regulatory networks. We identified a conserved set of pathways crucial for efflux activity in these experimental conditions, which expands the list of genes known to impact on efflux efficacy. Responses in both species were similar and we propose that these common results represent a core set of genes likely to be relevant to efflux control across the Enterobacteriaceae.

## Introduction

Bacteria have evolved many mechanisms to resist the action of antibiotics, including the use of efflux pumps. These membrane proteins are involved in the active transport of antimicrobials, metabolites and other compounds from the cell [1, 2]. Efflux pumps are ubiquitous in bacteria and are of particular clinical importance due to their role in conferring decreased susceptibility to a wide range of commonly used antibiotics [3, 4]. Antibiotic-resistant isolates are often found to contain mutations that increase the expression or alter the specificity of efflux pumps, thereby reducing the amount of antibiotic that reaches its target and increasing the chances of treatment failure [1, 3–5]. Efflux alone is often only responsible for a modest increase in the minimum inhibitory concentrations (MICs) of antibiotics, which are typically 2–8-fold higher in efflux overexpressing mutants than for susceptible strains. However, efflux acts as a platform for most other resistance mechanisms [6]. For example, target site mutations in *gyrA* in *Escherichia coli* confer resistance to fluoroquinolones, but these mutant cells become susceptible when efflux is inactivated [7, 8]. The most clinically important group of multidrug resistance efflux pumps in Enterobacteriaceae is the resistance nodulation division (RND) family, with the best studied being the AcrAB–TolC system [3]. Homologues of AcrAB–TolC have been identified in most commensal and pathogenic Gram-negative bacteria [9]. In *E. coli *, *acrAB* are negatively regulated by the locally encoded repressor AcrR and positively regulated by the homologous global transcriptional activators MarA and SoxS (with an additional homologue, RamA, present in *Salmonella enterica* serovar Typhimurium and various other Enterobacteriaceae, but not *E. coli *) [10–12]. Expression of *marA*, *ramA* and *soxS* is negatively regulated by their own local repressors MarR, RamR and SoxR, which is ablated by inducers, which often include substrates of AcrAB–TolC [11]. As well as impacting on expression of *acrAB*, MarA, RamA and SoxS can also regulate the expression of other efflux pumps, including the RND pump AcrEF [5] and a member of the multidrug and toxic compound extrusion (MATE) family, MdtK [13] in response to environmental stress [11].

Whilst the role of mutation of local repressors [14] and global activators such as Mar/Sox/Ram [11] has been well studied in relation to efflux control, many other pathways and gene products have been implicated as playing a role in efflux function [1, 15]. The bacterial cell is an interconnected metabolic network and many genes in different pathways have been proposed to impact on efflux activity or function. This suggests that efflux activity is deeply interconnected with many fundamental cellular processes. To further understanding of how efflux activity is controlled and how this impacts on the bacterial host cell requires a genome-wide approach. Large-scale genome-wide screens have previously proven effective in identifying repertoires of genes involved in survival and fitness in defined stress conditions: One such technique is TraDIS-*Xpress*, which uses massive transposon mutant libraries where each cell contains a randomly inserted transposon including an outwards-transcribing inducible promoter [16]. This allows the impact of all genes within a cell on survival in the test condition to be measured simultaneously in parallel. TraDIS-*Xpress* includes alteration of expression of genes immediately adjacent to transposon insertions, providing information on how expression changes affect fitness, as well as allowing the study of the impact of gene inactivation. We have recently used this method to identify genes involved in survival when exposed to several antimicrobials of different classes [16–19], as well as identifying novel antimicrobial combinations [20] and determining the genes involved in biofilm development over time in *E. coli* [21, 22].

Here we used TraDIS-*Xpress* to identify genes influencing efflux activity. We exposed transposon mutant libraries of both *E. coli* and *S*. Typhimurium to the canonical efflux substrate acriflavine (after which the AcrAB efflux system was named) [23]. Acriflavine is toxic and efflux is the known main mechanism of resistance in Gram-negative bacteria. To cleanly identify genes required for efflux expression rather than any drug-specific impacts, experiments were repeated in the presence and absence of the efflux inhibitor phenylalanine-arginine β-naphthylamide (PAβN). This approach allowed us to identify genes specifically involved in maintaining efflux function. We identified known efflux systems and regulators of efflux activity, confirming the specificity of the stress conditions, but also identified a wider repertoire of genes contributing to efflux function. This included genes with roles in cell envelope maintenance, stringent response activation, DNA housekeeping, respiration and glutathione biosynthesis. Comparing results from both *E. coli* and *S*. Typhimurium highlighted pathways conserved in both as well as some species-specific genes. These data expand our understanding of how efflux activity is maintained in important pathogens, revealing alternative pathways that may be exploited to ablate efflux activity as a strategy against multidrug resistance.

## Methods

### Transposon mutant library exposure conditions

The transposon mutant library in *E. coli* used in this study was described by Yasir *et al*. [16] and contains over 800 000 unique mutants, which roughly equates to an insertion every 6 bp. The transposon mutant library in *S*. Typhimurium described by Holden *et al*. [21] is also very dense, containing approximately 500 000 unique mutants and an insertion every 10 bp. Approximately 10^7^ c.f.u. ml^−1^ of transposon mutant library was added to 5 ml lysogeny broth (LB) in an untreated six-well plate (Sigma). The minimum inhibitory concentration (MIC) of acriflavine in the parent *E. coli* and *S*. Typhimurium strains was determined to be 256 µg ml^−1^ following the microbroth dilution method [24]. Conditions were either left untreated or were supplemented with subinhibitory (64 mg l^−1^) or inhibitory (256 µg ml^−1^) concentrations of acriflavine. Each condition was also prepared with or without a subinhibitory concentration of PAβN (125 µg ml^−1^) to elucidate genes involved in efflux specifically. Each condition was also prepared with and without 1 mM IPTG to induce expression from the transposon-located *tac* promoter, crucial for investigating how expression affects survival and for assaying essential genes. Two independent replicates were prepared for each condition. Plates were incubated at 30 °C with light agitation at 60 r.p.m. Following 24 h incubation, the cultures were centrifuged at 2100 ***g*** for 10 min to form pellets, from which genomic DNA was extracted following the protocol described by Trampari *et al*. [25]

### Sequencing library preparation and informatics

Sequencing libraries were prepared following the protocol outlined previously [16]. Genomic DNA was fragmented using the MuSeek DNA library preparation kit (Invitrogen) and purified with 1.5× volume of AMPure beads (Beckman Coulter). Fragments containing the transposon were amplified by PCR using customized primers specific for the transposon and for the MuSeek tags. The PCR products were cleaned twice with 0.6× volume and 1× volume of AMPure beads to purify and size-select fragments between 300–500 bp. Samples were sequenced on a NextSeq 500 using a NextSeq 500/550 High Output v2 kit (Illumina, 75 cycles). FastQ files were aligned to the *E. coli* (CP009273) and *S*. Typhimurium (CP001363, modified to include chromosomally integrated *lacIZ*) reference genomes using BioTraDIS (version 1.4.3) [26]. This produced plot files mapping the location and frequency of transposon insertions across the genome. Insertion frequencies were compared using the tradis_comparison.R command in the BioTraDIS toolkit, which determined the log fold change between insertions in the treated conditions relative to the controls. Significant differences were denoted with a *q*-value (*P*-value corrected for false discovery rate) of less than 0.05. AlbaTraDIS [27] was also used to determine significant differences between treated conditions and controls, identifying changes in insertion frequencies outside of coding regions associated with changes in gene expression due to the transposon-located promoter.

### Phenotypic investigations of efflux activity

TraDIS-*Xpress* results were validated by comparing the efflux activity of single-gene-deletion mutants to the wild-type. Gene deletion mutants in *E. coli* were selected from the Keio collection [28]. In *S*. Typhimurium, gene deletion mutants were constructed following the gene doctoring protocol [29] using tools constructed by golden gate assembly [30]. Constructs were confirmed by Sanger sequencing and whole-genome sequencing following the protocol described by Trampari *et al*. [25]. Efflux function was examined by measuring redox of resazurin dye over 60 min.

Resazurin is a non-fluorescent blue dye that undergoes an irreversible redox reaction and colorimetric change upon entering the cytosol. This colour change does not occur if the dye is removed from the cell via efflux before it is oxidized in the cytosol, making resazurin suitable as a dye to indicate intracellular accumulation. Colour changes over time were measured every 3 min for an hour in the presence and absence of the efflux inhibitor PAβN to determine efflux activity in knockout mutants relative to the wild-type. Bacteria were grown to mid-logarithmic growth phase and normalized to an OD_600nm_ of 0.1 in sterile phosphate-buffered saline (PBS). Resazurin dye and PAβN were added at a final concentration of 10 µg and 125 µg ml^−1^, respectively, to 200 µl of diluted culture. Fluorescence of resazurin (excitation=544 nm, emission=590 nm) was measured in a FLUOstar Omega plate reader (BMG Labtech) and normalized using OD measurements of bacterial cell density. Experiments included three biological replicates for all samples and were repeated on separate occasions. The area under the curve was calculated using the AUC function from the DescTools package (version 0.99.28) in R (version 4.1.1). Conditions with and without PAβN were compared to differentiate between where genes affected efflux activity or membrane permeability.

Efflux function was also determined by measuring susceptibility to known efflux substrates [25]. The MICs of acriflavine, azithromycin, cefotaxime and gentamycin (a non-efflux substrate control) were determined following the microbroth dilution method [24] using two biological and two technical replicates.

## Results

### TraDIS-*Xpress* identified multiple pathways that affect efflux activity in both species

*E. coli* and *S*. Typhimurium transposon mutant libraries were grown in subinhibitory and inhibitory concentrations of acriflavine and differences in the abundance of mutants across the genome in each condition were identified by comparison to unstressed controls. Predictions as to genes where inactivation or altered expression had a role in survival were then made. To identify genes specifically involved in efflux activity from those involved in acriflavine susceptibility, experiments were replicated in the presence and absence of the efflux inhibitor PAβN and the results were then compared against exposure to acriflavine alone and PAβN alone (to control for any intrinsic activity of the inhibitor alone). Altogether, 66 genes in *E. coli* and 94 genes in *S*. Typhimurium were found to affect survival in any condition – in the presence of acriflavine, PAβN, or a combination of the two (Table S1, available in the online version of this article). Differences in insertion frequencies per gene between replicates was low, indicating low experimental error (Fig. S1). Pathways involved in acriflavine susceptibility alone (not efflux) were then disregarded to identify genes and pathways specific to efflux activity. This resulted in a total of 62 genes confidently predicted to impact on efflux activity across both species: 37 in *E. coli* and 34 in *S*. Typhimurium (Table S1).

**Fig. 1.**
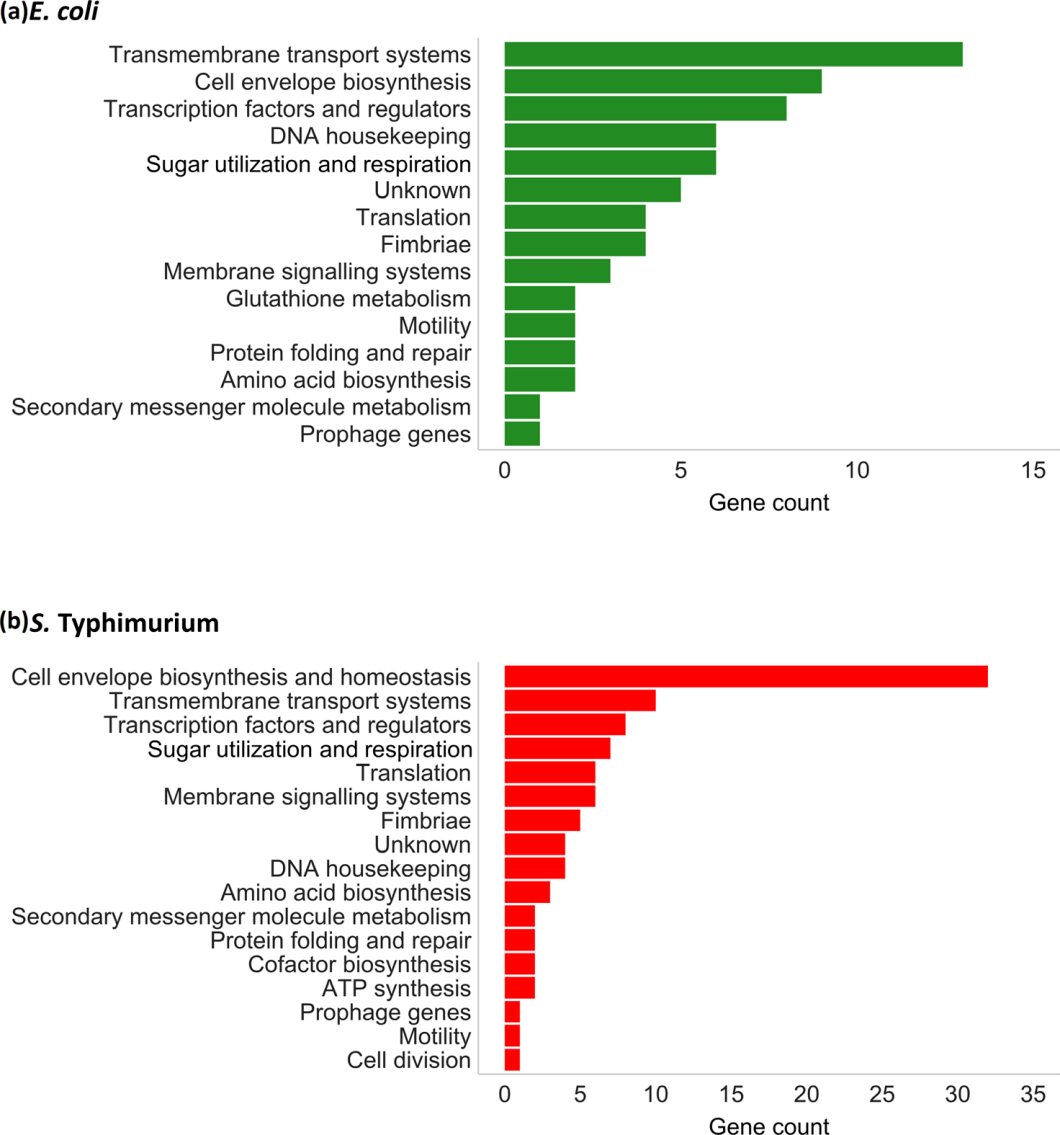
Analysis of the pathways involved in efflux activity and acriflavine susceptibility in *E. coli* (green) and *S*. Typhimurium (red).

Genes predicted to affect efflux activity had diverse roles belonging to many pathways, including ribosome modification, respiration, glutathione metabolism, DNA housekeeping, cell signalling, transcriptional regulation and chaperoning of proteins (Fig. 1). For most of these functional groups similar numbers of genes were found in both species, except for genes involved in cell envelope biosynthesis, where more hits were found in *S*. Typhimurium relative to *E. coli *. However, this is most probably due to known LPS deficiencies in K-12 strains of *E. coli* such as BW25113 used in this study [31]. To investigate how these genes affected efflux activity, antimicrobial susceptibility and dye accumulation was measured in defined deletion mutants in the presence and absence of PAβN (Fig. S2). Antimicrobial susceptibility was tested against acriflavine, azithromycin and cefotaxime, all of which are substrates of RND efflux pumps, and gentamycin (included as a poorly effluxed control substrate). Single-gene-deletion mutants in *E. coli* were retrieved from the Keio collection [28] and several new deletion mutants in *S*. Typhimurium were constructed for this study. Genes that appeared to affect efflux in both species were validated using *E. coli* mutants alone.

### AcrAB and regulators had the strongest impact on efflux activity

**Fig. 2.**
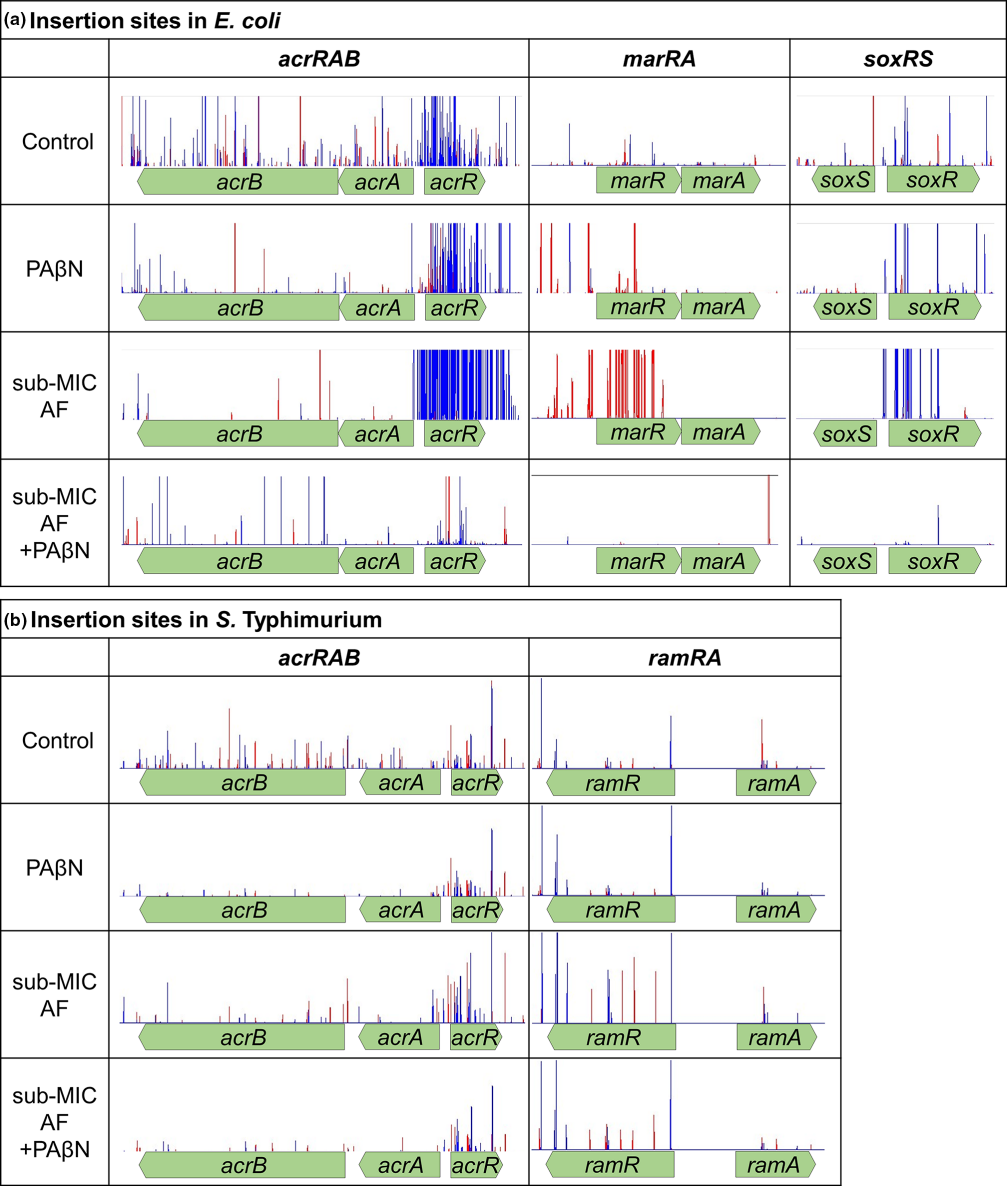
Transposon insertion sites in (a) *acrRAB*, *marRA* and *soxRS* in *E. coli* and (b) *acrRAB* and *ramRA* in *S*. Typhimurium in untreated controls, with PAβN, with subinhibitory concentrations of acriflavine (AF), and the two compounds combined. Insertion frequencies are shown by the height of the peak and the colour represents the direction of the outward-transcribing promoter. Images are representative of two independent replicates.

The largest differences in insertion frequencies between the stress conditions and controls were in genes encoding known efflux systems and their regulators, validating the specificity of the experimental setup for efflux. In both *E. coli* and *S*. Typhimurium, significantly fewer insertions mapped to *acrA*, *acrB* and *tolC* in conditions stressed with acriflavine or PAβN relative to unstressed controls (Fig. 2a, b), indicating that these genes are beneficial for efflux activity. In the same conditions, there were more insertions in the local regulator of *acrAB* expression, *acrR*, demonstrating its negative effect on efflux activity (Fig. 2a, b). There was a 12.1 log fold difference in the number of insertions within *acrR* between the control and acriflavine conditions in *E. coli *, further confirming the specificity of this condition in identifying genes involved in efflux activity. No significant signal was seen for *envR*, another repressor of *acrAB*, and the impact of loss of envR may be compensated for by *acrR* repression.

**Fig. 3.**
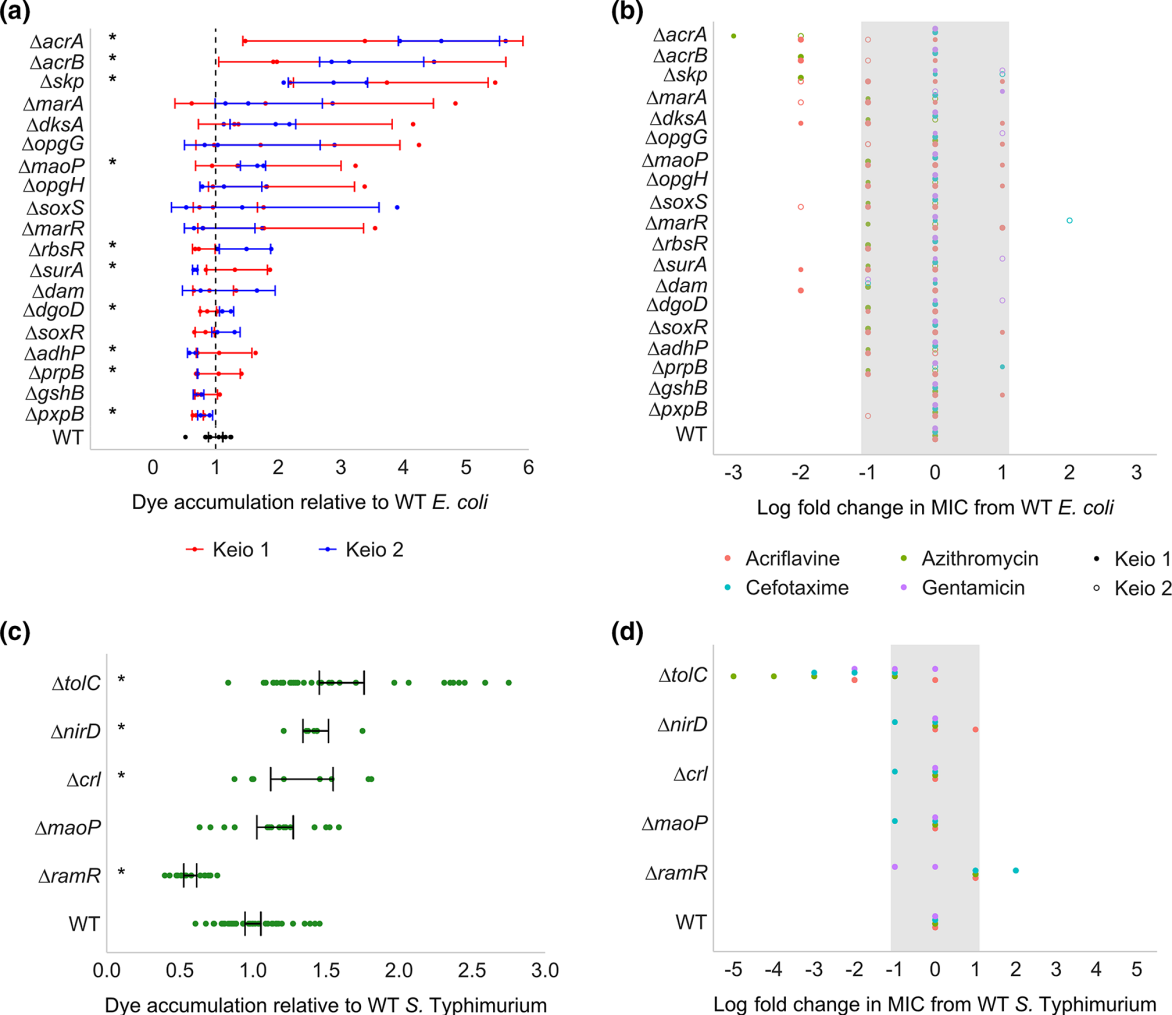
Relative efflux activity in single-gene-deletion mutants. (**a**) Relative accumulation of resazurin dye between wild-type *E. coli* and gene deletion mutants. (**b**) Log fold change in antimicrobial MICs of gene deletion mutants relative to wild-type *E. coli *. For both plots, the two copies of each mutant from the Keio collection were analysed separately. (**c**) Relative accumulation of resazurin dye between wild-type *S*. Typhimurium. (**d**) Log fold change in antimicrobial MICs of gene deletion mutants relative to wild-type *S*. Typhimurium. For both dye accumulation graphs, accumulation was measured over 60 min in a minimum of three independent replicates with and without PAβN and the area under the curve was calculated. Error bars show 95 % confidence intervals. Significant differences (Welch’s *t*-test) in accumulation between the wild-type and gene deletion mutants are shown with asterisks (*). For both MIC graphs, points show two independent replicates, and the grey shaded area shows the error of the test, 1 log fold change.

The global regulators MarA and SoxS (and RamA in *S*. Typhimurium) were also shown to have a strong effect on efflux activity. TraDIS-*Xpress* data show that increased expression of both *marA* and *soxS*, as well as insertional inactivation of their local regulators *marR* and *soxR*, was beneficial for efflux activity in *E. coli* (Fig. 2a). Confirming these findings, deletion of *marA* or *soxS* in *E. coli* resulted in increased acriflavine susceptibility, and deletion of the negative regulator *marR* reduced cefotaxime susceptibility (Fig. 3b). There was no significant change in dye uptake following deletion of either *marA* or *soxS* compared to the parent, which is consistent with the need for these genes to be overexpressed to exert an impact on the functional redundancy reported between these regulators [11]. In *S*. Typhimurium, deletion of *ramR* was beneficial for efflux activity (Fig. 2b, Fig. 3c, d), but no significant changes were seen in *marRA* or *soxRS*. This supports previous work suggesting a difference in the hierarchy of importance of these regulators between each species, even though all mutants would have been present in the pool [11].

Beyond AcrAB–TolC, other transmembrane transport systems were also identified by TraDIS-*Xpress* to benefit efflux in *E. coli* and *S*. Typhimurium. These included *gltS* encoding a glutamate : sodium symporter [32], *satP* encoding an acetate/succinate:H^+^ symporter [33], and *osmF* [34], *potA* and *gltJ* [35], each encoding components of different ABC transport systems and all affecting efflux in *E. coli* (Fig. 3a, b). The *S*. Typhimurium genome contains another efflux pump important for acriflavine resistance, encoded by *smvA* [36], which is not present in *E. coli *. TraDIS-*Xpress* also identified *smvA* to be strongly beneficial for the efflux of acriflavine in *S*. Typhimurium. This was the most prominent species-specific difference in transport systems identified between *E. coli* and *S*. Typhimurium, suggesting the importance of these wider transporters is relatively similar across Enterobacteriaceae.

### Activation of stress response mechanisms positively affect efflux activity

Bacteria can adapt quickly to environmental changes by invoking stress responses that involve various transcriptional regulators, which alter expression of genes to help alleviate the stress in question [37]. The stringent response is activated by signals of nutrient starvation and is regulated by transcription factors *dksA* and *rpoS* [38]. TraDIS-*Xpress* found *dksA* to be required for efficient efflux in *E. coli* and the same for *rpoS* in *S*. Typhimurium. Supporting this, deletion of *dskA* in *E. coli* resulted in a significant increase in acriflavine susceptibility (Fig. 3b), however previous work found no significant difference in efflux activity following deletion of *rpoS* in S. Typhimurium [39]. These genes may affect efflux regulation through *marR*, whose expression has been reported to increase (resulting in repression of *marA*) following deletion of either *dksA* [40] or *rpoS* [41]. We also identified two other genes that benefit efflux activity in *S*. Typhimurium that have a direct positive effect on *rpoS* transcription and activity: *iraP* [42] and *crl* [43]. We found that deletion of *crl* in *S*. Typhimurium resulted in increased accumulation of efflux substrates (Fig. 3c), indicative of reduced efflux activity, supporting the findings of the TraDIS-*Xpress* data.

Two-component signalling systems comprise a membrane-bound histidine kinase for sensing stress in the periplasm and a transcriptional regulator in the cytoplasm that raises a response. Our findings suggest that the two-component systems PhoPQ and CpxAR control efflux-related stress responses. Both *phoP* and *phoQ* benefitted efflux activity in *S*. Typhimurium treated with PAβN, suggesting that this system is beneficial for survival when efflux is inhibited. Conversely, *cpxA*, encoding the sensory kinase component of the CpxAR two-component system, was found to be detrimental for efflux activity in *S*. Typhimurium. However, these results clash with previous work describing a role for CpxA in positively regulating *marA* and *tolC* transcription [44] and evidence that CpxA is important for survival when TolC-dependent efflux is inhibited [45]. Our results may show a substrate-specific effect of CpxA, demonstrating the complex relationship between two-component signalling systems and efflux activity.

The activity of OpgG and OpgH, which produce periplasmic glucans implicated in osmotic regulation [46], seemed to negatively affect efflux activity in both *E. coli* and *S*. Typhimurium, according to the TraDIS-*Xpress* data. Deletion of either *opgG* or *opgH* did not, however, result in a significant change in dye uptake or drug susceptibility of mutant *E. coli* (Fig. 3a, b). Although the exact role of osmoregulated periplasmic glucans has not been elucidated, it has been suggested that they have a role in signalling, whereby their concentration in the periplasm may affect the activity of the EnvZ-OmpR and RcsCDB signalling systems [46, 47].

### Cell envelope maintenance modulates efflux activity in *E. coli* and *S*. Typhimurium

Chaperones involved in translocation and folding of membrane proteins had a considerable effect on efflux activity. We identified that both *skp* and *surA* had a negative effect on efflux activity in *E. coli *. Deletion of *surA* significantly reduced dye uptake in *E. coli* (Fig. 3a), in accordance with the predictions made by TraDIS-*Xpress*. However, contrary to the TraDIS-*Xpress* data, deletion of *skp* significantly increased dye uptake in *E. coli* (Fig. 3a). In the presence of the efflux inhibitor PAβN, dye accumulation in a Δ*skp* mutant was reduced relative to the wild-type and unchanged in a Δ*surA* mutant (Fig. S2), suggesting that *skp* is likely to affect efflux and *surA* membrane permeability in an efflux-independent manner. In *S*. Typhimurium, TraDIS-*Xpress* found that efflux activity was negatively affected by *secB*, involved in the correct folding of outer membrane proteins, including TolC and the porin OmpF [48]. We also identified a positive effect for *degS* on efflux activity in *S*. Typhimurium. DegS is an inner membrane protease involved in the activation of σ^E^, which governs the expression of genes involved in protein chaperoning (such as *skp*), LPS biosynthesis and the envelope stress response [49], and therefore it is plausible that DegS affects efflux activity through this pathway.

### DNA modification, replication and respiration are all beneficial for efflux activity

Two genes involved in DNA modification and replication were predicted by TraDIS-*Xpress* to benefit efflux activity. In both *E. coli* and *S*. Typhimurium we found that *dam*, encoding DNA adenine methyltransferase, was beneficial for efflux activity. In *E. coli *, *dam* was only beneficial to fitness in conditions with functional efflux. In support of this, deletion of *dam* resulted in increased acriflavine susceptibility. However, deletion of *dam* did not significantly affect dye uptake in *E. coli *, therefore *dam* may affect acriflavine susceptibility independently from efflux. Multiple studies have suggested a link between *dam* and efflux activity [50, 51], but a regulatory relationship has not yet been characterized. Previous work reported increased expression of *rpoS* and *marR* in a *dam* mutant relative to the wild-type, but no change in expression of *acrAB*, *tolC* or *marA* [52]. However, other studies have reported conflicting results, such as reduced expression of *marR* [53] and increased expression of *marA* [54] following deletion of *dam*. If DAM methylation affects expression of the *mar* operon, a phenotypic change in dye uptake may not be visible due to redundancy between the MarA, RamA and SoxS regulons [11]. DAM methylation regulates the expression of genes in the SOS regulon involved in DNA repair [53, 55, 56] and acriflavine is known to bind to DNA [57] therefore it is possible that DAM methylation may protect DNA from acriflavine-mediated damage independently from efflux.

*MaoP*, involved in chromosome organisation and orientation [58], was also beneficial for efflux activity in *E. coli *: its deletion resulted in a significant increase in dye accumulation (Fig. 3a) but no change in drug susceptibility (Fig. 3b). Dye uptake or drug susceptibility did not change following deletion of *maoP* in *S*. Typhimurium (Fig. 3c, d). Previous work has found reduced expression of *maoP* in an *acrB*-deficient mutant relative to wild-type *E. coli* [59], further supporting the idea that *maoP* expression and efflux activity may be linked.

Genes involved in sugar degradation and respiration were beneficial for survival in *E. coli* and *S*. Typhimurium when under efflux stress. These include *dgoD*, involved in d-galactonate degradation [60], alcohol dehydrogenase *adhP* involved in mixed acid fermentation [61] and *prpB* involved in catabolism of propionate to pyruvate [62]. Supporting the TraDIS-*Xpress* findings, there was a significant decrease in dye accumulation in Δ*adhP* and Δ*prpB* mutants in *E. coli *, but a significant increase in a Δ*dgoD* mutant (Fig. 3a). In *S*. Typhimurium, *nirD*, a nitrite reductase involved in anaerobic growth [63], was also found to be beneficial. Dye uptake was significantly increased in a *nirD* mutant relative to the wild-type (Fig. 3c), supporting this finding. Both *adhP* and *nirD* have a defined relationship with efflux regulator RamA: RamA has been shown to directly bind to and increase transcription of *adhP* in *Klebsiella pneumoniae* [64]. Expression of *nirD* was reduced when *ramA* was overexpressed in *K. pneumoniae* [64] and when *acrB* was disrupted in *S*. Typhimurium (which leads to *ramA* overexpression) [15]. Sugar metabolism and respiration involve complex networks of interacting genes that affect mutants’ growth rates [65], which may explain differences in findings between isogenic mutant populations compared to data from competitive TraDIS-*Xpress* pooled libraries. Whilst links to efflux regulators have been suggested, how these genes impact on efflux activity is unclear from this regulatory relationship alone. Respiration generates ATP and a proton gradient to power efflux pumps, which may explain how these genes influence efflux activity.

### Accumulation of glutathione is detrimental to efflux activity

TraDIS-*Xpress* identified two genes involved in glutathione metabolism implicated in efflux activity in *E. coli *. Synthesis of glutathione via *gshB* [66] had a negative effect and the catabolism of glutathione to glutamate via *pxpB* had a positive effect on efflux activity [67]. This is supported by previous research suggesting that reduced periplasmic glutathione results in increased expression of *acrA*, *acrB* and *tolC* in *E. coli* [68]. Deletion of *gshB* or *pxpB* in *E. coli* did not result in a significant change in dye uptake or drug susceptibility (Fig. 3a, b), suggesting that the phenotypic impact from deletion of these genes may be mild.

## Discussion

**Fig. 4.**
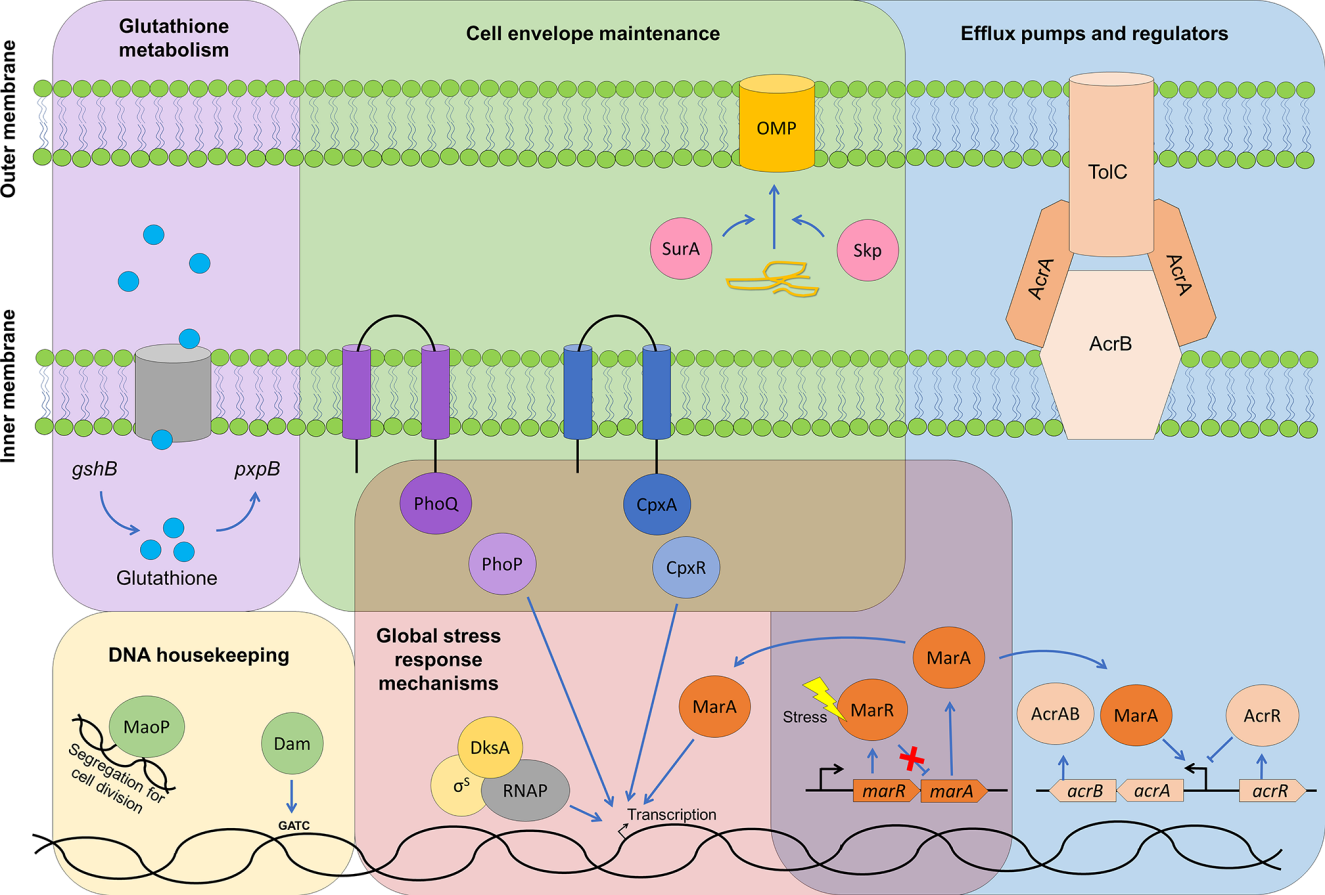
Summary of the pathways predicted to play a role in efflux activity in *E. coli* and *Salmonella *.

TraDIS-*Xpress* is a high-throughput genome-wide screening tool used to make predictions about the genes involved in survival in a stress condition. We designed experiments to use this method to determine the genes involved in efflux activity, using sub-inhibitory and inhibitory concentrations of the efflux substrate acriflavine in the presence or absence of the efflux inhibitor PAβN. This successfully identified known efflux systems and regulators, but also revealed roles for genes involved in cell envelope maintenance, DNA housekeeping, respiration and glutathione metabolism (Fig. 4). There was a high degree of similarity in the most important genes and pathways between *E. coli* and *S*. Typhimurium, consistent with the idea that regulation and control of efflux is an ancestral function that evolved to be efficient long ago.

Many of the genes and pathways identified in this genome-wide screen were found to have a direct or indirect effect on the expression of global efflux regulators MarA, RamA and SoxS. This demonstrates how involving efflux is a common part of a core stress response system that is triggered by all these systems. Interestingly, we only found an important role for RamA in *S*. Typhimurium, and mutants that overexpressed *marA* or *soxS* were not selected in efflux stress conditions. Previously, it has been observed that *Salmonella* appears to preferentially invoke efflux expression via the Ram system and our data support this assertion [69]. Mutants that had overproduced the other systems would have been present in the mutant pool, but this work suggests they may not have the fitness benefit expected. There may be more direct cross-talk between Mar/Ram/Sox in *Salmonella* than is currently understood, which prohibits Mar and Sox from achieving the same degree of impact as in *E. coli *.

For some genes, functional redundancy means that deletion of one will cause overexpression of others that will mask the phenotype, as is the case for MarA, RamA and SoxS. Whilst we used phenotypic validation of independent mutants to support many of the predictions made by the TraDIS-*Xpress* data, the phenotypic findings in single-gene-deletion mutants did not always support the prediction. Due to very dense transposon mutant libraries facilitating competition of each mutant against the pool, TraDIS-*Xpress* can capture very small changes in fitness and a gene is often implicated based on the change in abundance of dozens of independent mutants within the gene. The sensitivity of this approach means that the results cannot always be replicated in cruder whole-gene knockout mutants. This also explains why this study may not corroborate findings from previous studies using single knockout mutants, as differences in mutant fitness may not result in a large competitive advantage which can be readily measured in isogenic populations. Mutant libraries under competition are more representative of real-world environments where these bacteria and stresses may be found. Additionally, small changes in drug accumulation or drug sensitivity that may be measurable in a competition experiment may not be captured using the phenotypic methods used in this study, and further work would be needed to fully characterize the mechanisms by which all these genes affect efflux activity.

In summary, we have applied a genome-wide screen that identified known and new pathways that contribute to efflux function in two species. This revealed the deep connections of efflux with core cellular metabolism, with many different systems impacting on efflux function or regulation. We found some overlap with other important phenotypes, for example our recent work identified *maoP* and *dksA* as having important roles in biofilm formation and we also found these genes to be important for efflux [21]. These data reveal the connections present within the cell and highlight the enormously complex nature of the network of regulation and feedback mechanisms that interact to allow efflux to be used by a cell under attack. This work broadens our understanding of how efflux function is maintained within cells and paves the way for future studies using different efflux substrates and efflux inhibitors to provide a full view of how bacteria respond to efflux-related stresses. Comparing these results to other members of the Enterobacteriaceae family, as well as comparing different families of bacteria, would increase our understanding of the core pathways involved in efflux activity across important bacterial species. Further work is needed to understand how these interactions function and will allow a systems-based approach to model the control of efflux in infection relevant conditions.

## Supplementary Data

Supplementary material 1
