# Supplementary material for: Genome-wide analysis of genes involved in efflux function and regulation within Escherichia coli and Salmonella enterica serovar Typhimurium
Source: Microbiology (Reading). 2023 Feb 6;169(2):001296. doi: 10.1099/mic.0.001296 (PMC10197877; doi:10.1099/mic.0.001296)
Supplement: Supplementary material 1 [file mic-169-1296-s001.pdf]

Supplementary Data

Supplementary table 1: Genes determined by TraDIS-*Xpress* to be important for efflux activity and acriflavine susceptibility in *E. coli* (in blue) and *S.* Typhimurium (STM, in red), and the phenotypes of deletion mutants relative to the wild type. Log-fold change (LogFC) between test vs control conditions are only shown for genes where there are differences in insertion frequency inside the coding region. Where the plot files generated by BioTraDIS show a difference in insertion frequency upstream or downstream of a gene, log-fold change cannot easily by quantified and therefore the effect has been described in the column titled ‘observed change’. Significant differences in insertion frequencies have been manually verified with the plot files generated by BioTraDIS. Asterisks (*) denote whether the gene is confidently predicted to affect efflux activity.

Pathway	Gene	Difference in insertions			Phenotype of knockout mutant relative to WT	Predicted efflux effect	Ref
		Condition	LogFC	Observed change			
Efflux systems and regulators	*acrR*	subMIC vs ctrl	12.1	More insertions	• Reduced dye uptake with PAβN<br>• Reduced MIC of acriflavine<br>• No change in MIC	*	(1)
			0.4	More insertions			
		subMIC vs subMIC+PAβN	10.1	More insertions			
		MIC vs ctrl	-1.4	Fewer insertions			
	acrA	PAβN vs ctrl	-3.3	Fewer insertions	• Increased dye uptake<br>• Reduced dye uptake with PAβN<br>• Reduced MIC of acriflavine and azithromycin	*	
			-3.7	Fewer insertions			
		subMIC vs ctrl	-2.2	Fewer insertions			
	acrB	PAβN vs ctrl	-2.4	Fewer insertions	• Increased dye uptake<br>• Reduced dye uptake with PAβN<br>• Reduced MIC of acriflavine and azithromycin	*	
			-3.4	Fewer insertions			
		subMIC vs ctrl	-2.7	Fewer insertions			
	tolC	PAβN vs ctrl	-3.2	Fewer insertions	• No change in dye uptake or MIC<br>• Increased dye uptake<br>• Reduced dye uptake with PAβN<br>• Reduced MIC of acriflavine, azithromycin and cefotaxime	*	(2)
			-1.9	Fewer insertions			
	marA	PAβN vs ctrl		Increased expression beneficial	• Reduced dye uptake with PAβN<br>• Reduced MIC of acriflavine	*	(3)
		subMIC vs ctrl	-2.1	Increased expression beneficial & fewer insertions			

	marR	PAβN vs ctrl	1.0	More insertions	• No change in dye uptake<br>• Increased MIC for cefotaxime	*	
		subMIC vs ctrl	10.0	More insertions			
		subMIC vs subMIC+PAβN	10.6	More insertions			
		MIC vs ctrl	-3.9	Fewer insertions			
		MIC vs MIC+ PAβN	-4.6	Fewer insertions			
	soxS	subMIC vs ctrl	-2.8	Increased expression beneficial & fewer insertions	• Reduced dye uptake with PAβN<br>• Reduced MIC of acriflavine	*	(4)
		MIC vs ctrl		Reduced expression beneficial			
	soxR	subMIC vs ctrl	10.5	More insertions	• No change in dye uptake or MIC	*	
		subMIC vs subMIC+PAβN	11.1	More insertions			
		MIC vs ctrl	-1	Fewer insertions			
	ramR	subMIC vs ctrl	2.0	More insertions	• Reduced dye uptake with & without PAβN<br>• Increased MIC for cefotaxime	*	(5)
	smvA	subMIC vs ctrl	-2.2	Increased expression beneficial & fewer insertions	• Not tested	*	(6)
Transmembrane transport systems	*ompC*	PAβN vs ctrl	2.4	More insertions	• No change in dye uptake or MIC		(7)
	gltS	subMIC vs ctrl	-1.6	Fewer insertions	• No change in dye uptake or MIC	*	(8)
		subMIC vs subMIC+PAβN	-1.8	Fewer insertions			
	gltJ	subMIC vs ctrl	-3.8	Fewer insertions	• Reduced dye uptake with PAβN<br>• No change in MIC	*	(9)
	potA	subMIC vs ctrl	-4.2	Fewer insertions	• No change in dye uptake or MIC	*	
	proW	MIC vs ctrl		Increased expression beneficial	• No change in dye uptake or MIC	*	
	osmF	subMIC vs ctrl	-3.4	Fewer insertions	• Reduced dye uptake with PAβN<br>• No change in MIC	*	(10)
		subMIC vs subMIC+PAβN	-5.7	Fewer insertions			
	satP	subMIC vs ctrl	-2.5	Fewer insertions	• Reduced dye uptake<br>• No change in MIC	*	(11)
	glpF	MIC vs ctrl	4.6	More insertions	• Reduced dye uptake	*	(12)

					• No change in MIC		
	pitA	subMIC vs ctrl	1.1	More insertions	• Not tested	*	(13)
	corA	subMIC vs ctrl	1.1	More insertions	• Not tested	*	(14)
	potD	MIC vs ctrl	-2.4	Fewer insertions	• Not tested	*	(15)
		MIC vs MIC+PAβN	-2.8	Fewer insertions			
	secM	MIC vs ctrl	-8.6	Fewer insertions	• Not tested	*	(16)
	ybiR	MIC vs ctrl	-1.6	Fewer insertions	• Not tested	*	(17)
Transcription factors and regulators	*dksA*	PAβN vs ctrl	-3.9	Fewer insertions	• Reduced dye uptake with PAβN<br>• Reduced MIC for acriflavine	*	(18)
		subMIC vs ctrl	-4.2	Fewer insertions			
	rpoS	PAβN vs ctrl	-2.7	Fewer insertions	• Not tested	*	(19)
		subMIC vs ctrl	-2.9	Fewer insertions			
	iraP	PAβN vs ctrl	-1.5	Fewer insertions	• Not tested	*	(20)
		subMIC vs ctrl	-2.4	Fewer insertions			
	crl	PAβN vs ctrl	-1.5	Fewer insertions	• Reduced dye uptake with PAβN<br>• No change in MIC	*	(21)
		subMIC vs ctrl	-2.1	Fewer insertions			
	hupA	PAβN vs ctrl	-0.9	Fewer insertions	• Not tested	*	(22)
	iolR	subMIC vs ctrl	-2	Fewer insertions	• Not tested		(23)
		MIC vs ctrl	-1.8	Fewer insertions			
	gadW	PAβN vs ctrl	0.8	More insertions	• No change in dye uptake or MIC		(24)
	gadY	subMIC vs subMIC+PAβN	3.2	More insertions	• Not tested		(25)
	ybdF	subMIC vs ctrl		Increased expression beneficial	• Not tested		(26)
	STM14_1969	MIC vs ctrl	-9.1	Fewer insertions	• Not tested	*	(27)
		MIC vs MIC+PAβN	-9.6	Fewer insertions			
Membrane signalling systems	*phoP*	PAβN vs ctrl	-1.9	Fewer insertions	• Not tested	*	(28)
	phoQ	PAβN vs ctrl	-1.3	Fewer insertions	• Not tested	*	
	cpxA	subMIC vs ctrl	0.7	More insertions	• Not tested	*	(29)
	opgG	PAβN vs ctrl	2.9	More insertions	• No change in dye uptake or MIC	*	(30)
		subMIC vs subMIC+PAβN	-1.2	Fewer insertions			
	opgH	PAβN vs ctrl	1.5	More insertions	• No change in dye uptake or MIC	*	
		subMIC vs subMIC+PAβN	-1.5	Fewer insertions			
	ompR	subMIC vs ctrl	6.9	More insertions	• Reduced dye uptake	*	(7)

		subMIC vs subMIC+PAβN	2.8	More insertions	• Increased MIC for cefotaxime		
	arcA	subMIC vs subMIC+PAβN	-2.2	Fewer insertions	• Not tested	*	(31)
Secondary messenger molecule metabolism	*pdeC*	MIC vs ctrl	1.2	More insertions	• Reduced dye uptake with PAβN<br>• No change in MIC		(32)
	pdeK	PAβN vs ctrl	-1.8	Fewer insertions	• Reduced dye uptake with PAβN<br>• No change in MIC		
	cyaA	subMIC vs ctrl	1.2	More insertions	• No change in dye uptake or MIC		(33)
Protein chaperones and folding	*skp*	PAβN vs ctrl	4.1	More insertions	• Increased dye uptake<br>• Reduced dye uptake with PAβN<br>• Reduced MIC for acriflavine and azithromycin	*	(34)
	surA	subMIC vs ctrl	6.5	More insertions	• Reduced dye uptake<br>• Reduced MIC for acriflavine	*	(35)
	secB	PAβN vs ctrl	1.6	More insertions	• Not tested	*	(36)
		subMIC vs ctrl	2.1	More insertions			
	degS	PAβN vs ctrl	-1.1	Fewer insertions	• Not tested	*	(37)
DNA housekeeping	*dam*	subMIC vs ctrl	-1.4	Fewer insertions	• No change in dye uptake<br>• Reduced MIC for acriflavine	*	(38)
			-2.2	Fewer insertions			
		subMIC vs subMIC+PAβN	-3.8	Fewer insertions			
	maoP	PAβN vs ctrl	-5.4	Fewer insertions	• Increased dye uptake<br>• Reduced dye uptake with PAβN<br>• No change in MIC<br>• No change in dye uptake or MIC	*	(39)
	rep	PAβN vs ctrl	2.4	More insertions	• No change in dye uptake or MIC		(40)
	ybiB	PAβN vs ctrl	-1.4	Fewer insertions	• No change in dye uptake or MIC		(41)
	yjhQ	subMIC vs subMIC+PAβN	4.6	More insertions	• Reduced dye uptake with PAβN<br>• No change in MIC		(42)
	tehB	MIC vs MIC+PAβN	2	More insertions	• No change in dye uptake<br>• Reduced MIC for acriflavine		(43)
	dinI	MIC vs ctrl	-2.9	Fewer insertions	• Not tested		(44)
	recB	MIC vs ctrl	2.6	More insertions	• Not tested		(45)
	recC	subMIC vs ctrl	1.1	More insertions			

		MIC vs ctrl	2.5	More insertions	• Not tested		
Sugar utilisation and respiration	*dgoD*	subMIC vs ctrl	-0.3	Fewer insertions	• Increased dye uptake<br>• No change in MIC	*	(46)
		subMIC vs subMIC+PAβN	-1.3	Fewer insertions			
	prpB	MIC vs ctrl	-3.9	Fewer insertions	• Reduced dye uptake	*	(47)
		MIC vs MIC+PAβN	-2.1	Fewer insertions	• No change in MIC		
	adhP	subMIC vs ctrl	-2.5	Fewer insertions	• Reduced dye uptake<br>• No change in MIC	*	(48)
		subMIC vs subMIC+PAβN	-2.8	Fewer insertions			
	mhpF	subMIC vs ctrl	-4.2	Fewer insertions	• Reduced dye uptake with PAβN<br>• No change in MIC	*	(49)
	glk	MIC vs MIC+PAβN	5.1	More insertions	• Reduced dye uptake with PAβN<br>• No change in MIC	*	(50)
	rbsR	subMIC vs ctrl	-1.7	Fewer insertions	• Reduced dye uptake with or without PAβN<br>• No change in MIC	*	(51)
		subMIC vs subMIC+PAβN	-1.4	Fewer insertions			
	rbsK	subMIC vs ctrl		Increased expression beneficial	• Not tested	*	
	yhdA/ ydiV/ csrD	subMIC vs ctrl	0.7	More insertions	• Not tested		(32)
	nagA	PAβN vs ctrl	-2.7	Fewer insertions	• Not tested	*	(52)
	eutN	MIC vs ctrl	-2.6	Fewer insertions	• Not tested	*	(53)
	nirD	MIC vs ctrl	-2.7	Fewer insertions	• Increased dye uptake<br>• Reduced dye uptake with PAβN<br>• No change in MIC	*	(54)
	citG	MIC vs ctrl	-2.2	Fewer insertions	• Not tested	*	(55)
		MIC vs MIC+PAβN	-2.5	Fewer insertions			
	STM14_0712	MIC vs ctrl	-1	Fewer insertions	• Not tested	*	(56)
		MIC vs MIC+PAβN	-0.9	Fewer insertions			
Glutathione metabolism	*gshB*	PAβN vs ctrl	1.9	More insertions	• No change in dye uptake or MIC	*	(57)
	pxpB	subMIC vs ctrl	-4.3	Fewer insertions	• Reduced dye uptake with PAβN	*	(58)
		subMIC vs subMIC+PAβN	-2.6	Fewer insertions	• No change in MIC		
Amino acid biosynthesis	*metL*	MIC vs ctrl	2.5	More insertions	• Reduced dye uptake with & without PAβN<br>• No change in MIC	*	(59)
		PAβN vs ctrl	0.7	More insertions			

	leuL	subMIC vs ctrl	-0.1	Fewer insertions	• No change in dye uptake or MIC		(60)
	leuD	MIC vs ctrl	-1.1	Fewer insertions	• Not tested		
	argG	subMIC vs ctrl	0.6	More insertions	• Not tested		(61)
Cell envelope biosynthesis	*mrcA*	PAβN vs ctrl	-2.9	Fewer insertions	• Reduced dye uptake with PAβN<br>• No change in MIC		(62)
	mrcB	PAβN vs ctrl	-1.5	Fewer insertions	• Not tested		
	lpoA	PAβN vs ctrl	-3.8	Fewer insertions	• Reduced dye uptake with PAβN<br>• Reduced MIC for acriflavine		(63)
	ddlB	MIC vs ctrl	6.6	More insertions	• Not tested		(64)
	prc	MIC vs ctrl	2.5	More insertions	• Not tested		(65)
	ldcA	PAβN vs ctrl	-1.6	Fewer insertions	• Not tested		(66)
	nlpD	PAβN vs ctrl	-1.5	Fewer insertions	• Not tested		(67)
		subMIC vs ctrl	-1.8	Fewer insertions			
	cvpA	PAβN vs ctrl	-2.3	Fewer insertions	• Not tested		(68)
	tolB	subMIC vs ctrl	8.3	More insertions	• Not tested		(69)
		MIC vs ctrl	12.2	More insertions			
	tolQ	MIC vs ctrl	2.7	More insertions	• Not tested		
	tolA	MIC vs ctrl	2.3	More insertions	• Not tested		
	tolR	MIC vs ctrl	10.4	More insertions	• Not tested		
Enterobacterial common antigen	*wecF*	subMIC vs ctrl	5.9	More insertions	• Reduced dye uptake with & without PAβN<br>• No change in MIC		(70)
		subMIC vs ctrl	0.3	More insertions			
	wecA	PAβN vs ctrl	-2.3	Fewer insertions	• Not tested		
	wecB	PAβN vs ctrl	-2.2	Fewer insertions	• Not tested		
	wecC	PAβN vs ctrl	-2.6	Fewer insertions	• Not tested		
		subMIC vs ctrl	0.7	More insertions			
	wecG	PAβN vs ctrl	-1.4	Fewer insertions	• Not tested		
	wecE	subMIC vs ctrl	1.1	More insertions	• Not tested		
	wzxE	subMIC vs ctrl	0.8	More insertions	• Not tested		(71)
		MIC vs ctrl	3	More insertions			
	yhdP	PAβN vs ctrl	-1.2	Fewer insertions	• Not tested		(72)
LPS	*wzzB*	subMIC vs ctrl	-4.3	Fewer insertions	• Reduced dye uptake with & without PAβN		(73)
		subMIC vs subMIC+PAβN	-7.2	Fewer insertions	• No change in MIC		

	lpxD	PAβN vs ctrl		Increased expression beneficial	• Not tested		(74)
	waaP/ rfaP	PAβN vs ctrl	2	More insertions	• No change in dye uptake<br>• Reduced MIC for azithromycin<br>• Increased MIC for cefotaxime		(75)
	waaG/ rfaG	MIC vs ctrl	2.3	More insertions	• Increased dye uptake<br>• Reduced MIC for azithromycin<br>• Increased MIC for cefotaxime		
	waaF/ rfaF	subMIC vs subMIC+PAβN	-4	Fewer insertions	• No change in dye uptake<br>• Reduced MIC for azithromycin		
	rfaL	subMIC vs subMIC+PAβN	1.9	More insertions	• Not tested		
	rfaJ	subMIC vs subMIC+PAβN	1.6	More insertions	• Not tested		
	rfbH	PAβN vs ctrl	-4.7	Fewer insertions	• Not tested		
		subMIC vs ctrl	-4.4	Fewer insertions			
	rfbG	PAβN vs ctrl	-3.7	Fewer insertions	• Not tested		
		subMIC vs ctrl	-3.9	Fewer insertions			
	rfbF	PAβN vs ctrl	-5.3	Fewer insertions	• Reduced dye uptake dye uptake with & without PAβN<br>• Reduced MIC for gentamicin		
		subMIC vs ctrl	-4.4	Fewer insertions			
	rfbI	PAβN vs ctrl	-3	Fewer insertions	• Not tested		
		subMIC vs subMIC+PAβN	1.7	More insertions			
	rfbC	PAβN vs ctrl	-0.5	Fewer insertions	• Not tested		
		subMIC vs subMIC+PAβN	1.7	More insertions			
	rfbA	MIC vs ctrl	2.3	More insertions	• Not tested		
		subMIC vs subMIC+PAβN	1	More insertions			
	rfbK	MIC vs ctrl	1.2	More insertions	• Not tested		
	rfbM	MIC vs ctrl	1.7	More insertions	• Not tested		
	rfbN	subMIC vs subMIC+PAβN	1.5	More insertions	• Not tested		
	rfbU	MIC vs ctrl	3.2	More insertions	• Not tested		
	rfbX	MIC vs ctrl	6.3	More insertions	• Not tested		

	arnE	MIC vs MIC+PAβN	-3.6	Fewer insertions	• Not tested		(76)
Translation	*ychF*	subMIC vs ctrl	-2.4	Fewer insertions	• Increased dye up	*	(77)
Translation	*ychF*	subMIC vs subMIC+PAβN	-4.1	Fewer insertions	• No change in MIC	*	(77)
Translation	*rimK*	subMIC vs ctrl	-3.7	Fewer insertions	• No change in dye uptake or MIC		(78)
Translation	*ygaM*	subMIC vs ctrl	-3.6	Fewer insertions	• No change in dye uptake or MIC		(79)
Translation	*trhP*	subMIC vs ctrl	-2.1	Fewer insertions	• Reduced dye uptake with PAβN<br>• No change in MIC		(80)
Translation	*infB*	subMIC vs ctrl		Increased expression beneficial	• No change in dye uptake or MIC		(81)
Translation	*tuf_1*	subMIC vs ctrl	1.2	More insertions	• Not tested		(82)
Translation	*tuf_2*	subMIC vs ctrl	1.5	More insertions	• Not tested		(82)
Translation	*deaD*	subMIC vs ctrl	0.7	More insertions	• Not tested		(83, 84)
Translation	*bipA*	subMIC vs ctrl	0.9	More insertions	• Not tested		(85)
Translation	*proQ*	MIC vs ctrl	2	More insertions	• Not tested		(86)
Fimbriae	*fimB*	PAβN vs ctrl	0.8	More insertions	• Reduced dye uptake with PAβN<br>• No change in MIC		(87)
Fimbriae	*fimB*	MIC vs MIC+PAβN	0.6	More insertions	• Reduced dye uptake with PAβN<br>• No change in MIC		(87)
Fimbriae	*fimZ*	subMIC vs ctrl	0.5	More insertions	• Not tested		(88)
Fimbriae	*fimY*	subMIC vs ctrl	0.5	More insertions	• Not tested		(88)
Fimbriae	*fimE*	PAβN vs ctrl	0.6	More insertions	• Reduced dye uptake with PAβN<br>• No change in MIC		(87)
Fimbriae	*fimE*	subMIC vs ctrl	-2.2	Fewer insertions	• Reduced dye uptake with PAβN<br>• No change in MIC		(87)
Fimbriae	*fimE*	MIC vs ctrl	-1	Fewer insertions	• Reduced dye uptake with PAβN<br>• No change in MIC		(87)
Fimbriae	*fimW*	PAβN vs ctrl	-1.7	Fewer insertions	• Not tested		(88)
Fimbriae	*fimW*	subMIC vs ctrl	-1.8	Fewer insertions	• Not tested		(88)
Fimbriae	*fimW*	MIC vs ctrl	-1.9	Fewer insertions	• Not tested		(88)
Fimbriae	*fimC*	MIC vs ctrl	1	More insertions	• Not tested		(89)
Fimbriae	*fimD*	MIC vs ctrl	1.4	More insertions	• Not tested		(89)
Fimbriae	*fimF*	subMIC vs ctrl	0.5	More insertions	• Not tested		(89)
Fimbriae	*ppdC*	MIC vs ctrl	-3.3	Fewer insertions	• Not tested		(90)
Motility	*lrhA*	PAβN vs ctrl	-3	Fewer insertions	• No change in dye uptake or MIC		(91)
Motility	*lrhA*	subMIC vs ctrl	-3.2	Fewer insertions	• No change in dye uptake or MIC		(91)
Motility	*hdfR*	PAβN vs ctrl	-6.5	Fewer insertions	• No change in dye uptake or MIC		(92)
Motility	*hdfR*	subMIC vs ctrl	-3.5	Fewer insertions	• No change in dye uptake or MIC		(92)
ATP synthesis	*atpA*	MIC vs ctrl	3.5	More insertions	• Not tested		(93)

	atpG	MIC vs ctrl	3.1	More insertions	• Not tested		
Prophage genes	*yagL*	MIC vs ctrl		Reduced expression beneficial	• Increased dye uptake<br>• Reduced dye uptake with PAβN<br>• No change in MIC		(94)
	STM14_1418 - 1483 (Gifsy-3 prophage)	subMIC vs subMIC+PAβN	1.3 (mean)	Fewer insertions	• Not tested		(95)
Cofactor biosynthesis	*panD*	MIC vs ctrl	-2.1	Fewer insertions	• Not tested		(96)
	cbiT	MIC vs ctrl	-2.7	Fewer insertions	• Not tested		(97)
Cell division	*zapE*	subMIC vs ctrl	0.7	More insertions	• Reduced dye uptake with PAβN<br>• No change in MIC		(98)
Unknown	*ybaA*	subMIC vs ctrl		Increased expression beneficial	• Reduced dye uptake with PAβN<br>• No change in MIC		
	ybbC	MIC vs ctrl		Increased expression beneficial	• No change in dye uptake or MIC		
	ybjM	subMIC vs subMIC+PAβN	-8.5	Fewer insertions	• No change in dye uptake or MIC	*	
	yhcB	subMIC vs subMIC+PAβN	-4.4	Fewer insertions	• Reduced dye uptake with PAβN<br>• No change in MIC	*	
	ydeJ	subMIC vs subMIC+PAβN	-2.7	Fewer insertions	• No change in dye uptake or MIC	*	
	yfbU	MIC vs ctrl	-0.7	Fewer insertions	• Not tested		
	STM14_0777	MIC vs ctrl	-2.1	Fewer insertions	• Not tested		
	STM14_2429	MIC vs ctrl	-2	Fewer insertions	• Not tested		
	STM14_2263	subMIC vs subMIC+PAβN	-1.3	Fewer insertions	• Not tested	*	

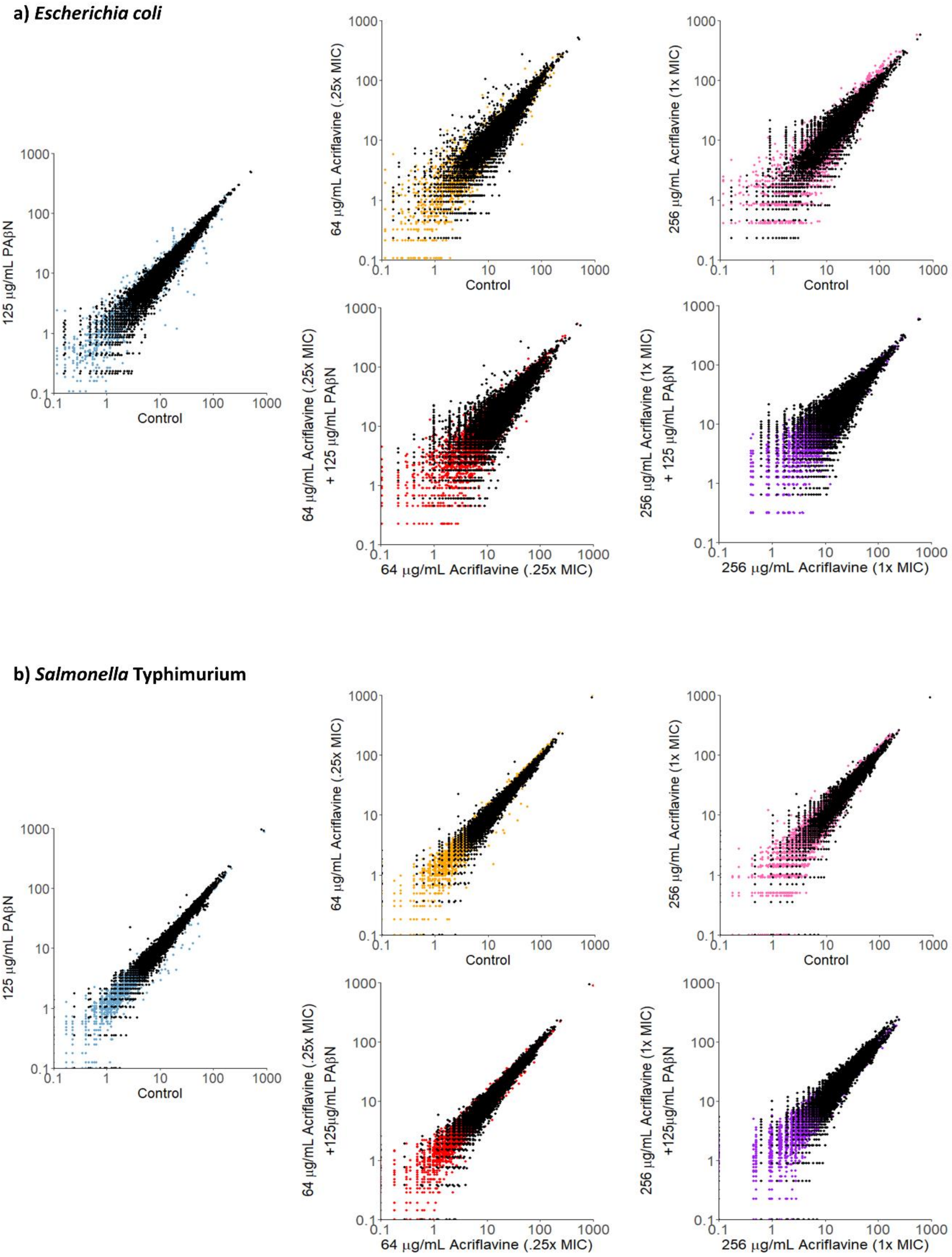

Supplementary figure 1: Insertion frequency per gene for the **a)** *E. coli* and **b)** *S*. Typhimurium transposon mutant libraries cultured under two concentrations of acriflavine (64 and 256 µg/mL) and/or in the presence and absence of the efflux inhibitor PAβN (125 µg/mL), relative to unstressed controls. Black points represent the insertion frequency per gene for each replicate to show variation between the replicates, and coloured points show the insertion frequency per gene of each condition.

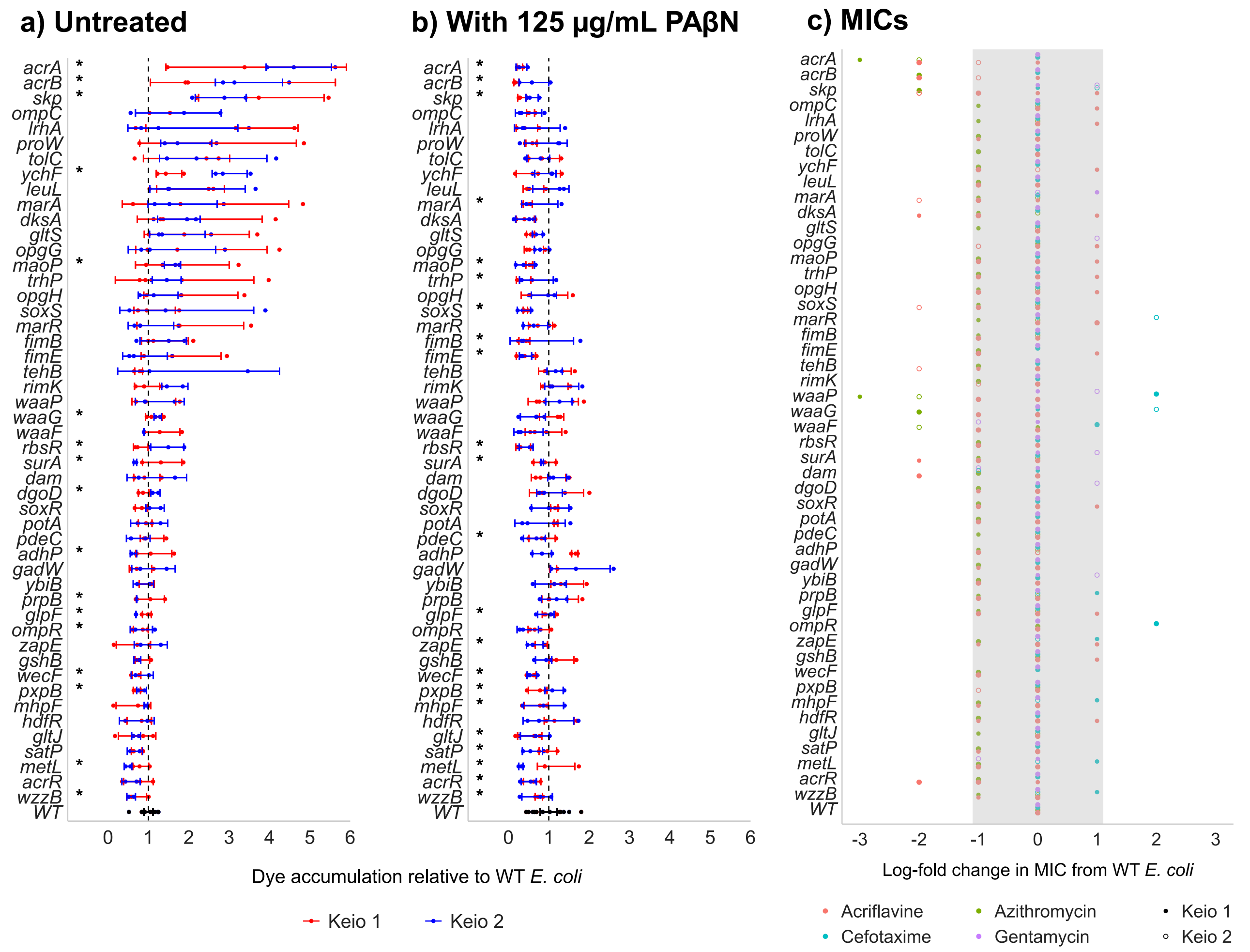

Supplementary figure 2: Efflux activity of single gene deletion mutants relative to wild type (WT) *E. coli*. **a)** Uptake of resazurin dye in gene deletion mutants relative to WT in unstressed conditions and **b)** with 125 μg/mL PAβN, as an indirect measurement of efflux activity. Dye accumulation was measured over 60 minutes in three independent replicates and the area under the curve was calculated. Error bars show 95% confidence intervals. Significant differences (Welch's *t*-test) in accumulation between the wild type and gene deletion mutants are shown with an asterisks (*). **c)** Log-fold change in antimicrobial MICs of gene deletion mutants relative to WT. Points show two independent replicates and the grey shaded area shows the error of the test, 1 log-fold change. For all plots, the two copies of each mutant from the Keio collection were analysed separately.

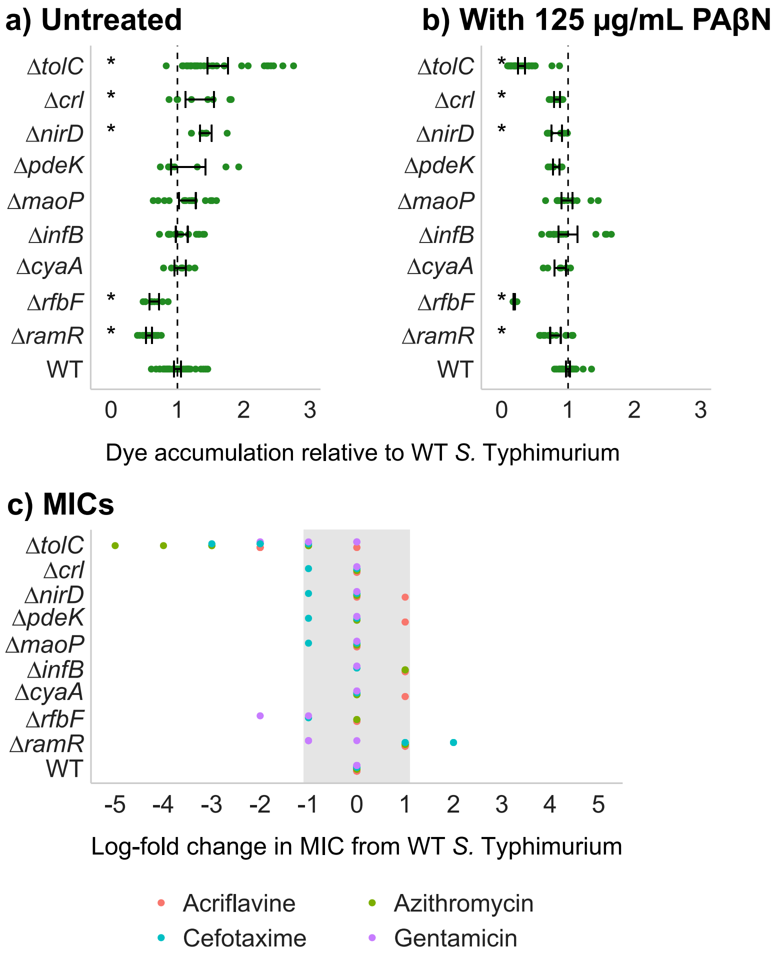

Supplementary figure 3: Efflux activity of single gene deletion mutants relative to wild type (WT) *S.* Typhimurium. **a)** Uptake of resazurin dye in gene deletion mutants relative to WT in unstressed conditions and **b)** with 125 µg/mL PAβN, as an indirect measurement of efflux activity. Dye accumulation was measured over 60 minutes in a minimum of three independent replicates and the area under the curve was calculated. Error bars show 95% confidence intervals. Significant differences (Welch's *t*-test) in accumulation between the wild type and gene deletion mutants are shown with an asterisks (*). **c)** Log-fold change in antimicrobial MICs of gene deletion mutants relative to WT. Points show two independent replicates and the grey shaded area shows the error of the test, 1 log-fold change.

References for supplementary material

1. Ma D, Cook DN, Alberti M, Pon NG, Nikaido H, Hearst JE. Genes *acrA* and *acrB* encode a stress-induced efflux system of *Escherichia coli*. Mol Microbiol. 1995;16(1):45-55.
2. Morona R, Manning PA, Reeves P. Identification and characterization of the TolC protein, an outer membrane protein from *Escherichia coli*. J Bacteriol. 1983;153(2):693-9.
3. Cohen SP, Hächler H, Levy SB. Genetic and functional analysis of the multiple antibiotic resistance (*mar*) locus in Escherichia coli. J Bacteriol. 1993;175(5):1484-92.
4. Demple B. Redox signaling and gene control in the *Escherichia coli soxRS* oxidative stress regulon — a review. Gene. 1996;179(1):53-7.
5. Abouzeed YM, Baucheron S, Cloeckaert A. *ramR* Mutations Involved in Efflux-Mediated Multidrug Resistance in *Salmonella enterica* Serovar Typhimurium. Antimicrob Agents Chemother. 2008;52(7):2428-34.
6. Villagra NA, Hidalgo AA, Santiviago CA, Saavedra CP, Mora GC. SmvA, and not AcrB, is the major efflux pump for acriflavine and related compounds in *Salmonella* enterica serovar Typhimurium. The Journal of antimicrobial chemotherapy. 2008;62(6):1273-6.
7. Cai SJ, Inouye M. EnvZ-OmpR interaction and osmoregulation in *Escherichia coli*. J Biol Chem. 2002;277(27):24155-61.
8. Kalman M, Gentry DR, Cashel M. Characterization of the *Escherichia coli* K12 *gltS* glutamate permease gene. Mol Gen Genet. 1991;225(3):379-86.
9. Moussatova A, Kandt C, O'Mara ML, Tieleman DP. ATP-binding cassette transporters in *Escherichia coli*. Biochim Biophys Acta. 2008;1778(9):1757-71.
10. Lang S, Cressatti M, Mendoza KE, Coumoundouros CN, Plater SM, Culham DE, et al. YehZYXW of *Escherichia coli* Is a Low-Affinity, Non-Osmoregulatory Betaine-Specific ABC Transporter. Biochemistry. 2015;54(37):5735-47.
11. Sá-Pessoa J, Paiva S, Ribas D, Silva IJ, Viegas SC, Arraiano CM, et al. SATP (YaaH), a succinate-acetate transporter protein in *Escherichia coli*. Biochem J. 2013;454(3):585-95.
12. Braun T, Philippsen A, Wirtz S, Borgnia MJ, Agre P, Kühlbrandt W, et al. The 3.7 A projection map of the glycerol facilitator GlpF: a variant of the aquaporin tetramer. EMBO Rep. 2000;1(2):183-9.
13. Harris RM, Webb DC, Howitt SM, Cox GB. Characterization of PitA and PitB from *Escherichia coli*. J Bacteriol. 2001;183(17):5008-14.
14. Hmiel SP, Snavely MD, Miller CG, Maguire ME. Magnesium transport in *Salmonella* typhimurium: characterization of magnesium influx and cloning of a transport gene. J Bacteriol. 1986;168(3):1444-50.
15. Kashiwagi K, Miyamoto S, Nukui E, Kobayashi H, Igarashi K. Functions of potA and potD proteins in spermidine-preferential uptake system in *Escherichia coli*. J Biol Chem. 1993;268(26):19358-63.
16. Sarker S, Oliver D. Critical regions of *secM* that control its translation and secretion and promote secretion-specific *secA* regulation. J Bacteriol. 2002;184(9):2360-9.
17. Kehres DG, Janakiraman A, Slauch J, M, Maguire ME. Regulation of *Salmonella enterica* Serovar Typhimurium mntH Transcription by H2O2, Fe2+, and Mn2+. J Bacteriol. 2002;184(12):3151-8.
18. Mallik P, Paul BJ, Rutherford ST, Gourse RL, Osuna R. DksA is required for growth phase-dependent regulation, growth rate-dependent control, and stringent control of *fis* expression in *Escherichia coli*. J Bacteriol. 2006;188(16):5775-82.
19. Gentry DR, Hernandez VJ, Nguyen LH, Jensen DB, Cashel M. Synthesis of the stationary-phase sigma factor σs is positively regulated by ppGpp. J Bacteriol. 1993;175(24):7982-9.
20. Girard ME, Gopalkrishnan S, Grace ED, Halliday JA, Gourse RL, Herman C. DksA and ppGpp Regulate the σS Stress Response by Activating Promoters for the Small RNA DsrA and the Anti-Adapter Protein IraP. J Bacteriol. 2018;200(2):e00463-17.
21. Bougdour A, Lelong C, Geiselmann J. Crl, a low temperature-induced protein in *Escherichia coli* that binds directly to the stationary phase sigma subunit of RNA polymerase. J Biol Chem. 2004;279(19):19540-50.
22. Oberto J, Nabti S, Jooste V, Mignot H, Rouviere-Yaniv J. The HU regulon is composed of genes responding to anaerobiosis, acid stress, high osmolarity and SOS induction. PLoS One. 2009;4(2):e4367-e.

23. Kröger C, Fuchs Thilo M. Characterization of the myo-Inositol Utilization Island of *Salmonella enterica* serovar Typhimurium. J Bacteriol. 2009;191(2):545-54.
24. Tucker DL, Tucker N, Ma Z, Foster JW, Miranda RL, Cohen PS, et al. Genes of the GadX-GadW regulon in *Escherichia coli*. J Bacteriol. 2003;185(10):3190-201.
25. Tramonti A, De Canio M, De Biase D. GadX/GadW-dependent regulation of the *Escherichia coli* acid fitness island: transcriptional control at the *gadY–gadW* divergent promoters and identification of four novel 42 bp GadX/GadW-specific binding sites. Mol Microbiol. 2008;70(4):965-82.
26. Rosenblum R, Khan E, Gonzalez G, Hasan R, Schneiders T. Genetic regulation of the *ramA* locus and its expression in clinical isolates of *Klebsiella pneumoniae*. Int J Antimicrob Agents. 2011;38(1):39-45.
27. Herring CD, Blattner FR. Global transcriptional effects of a suppressor tRNA and the inactivation of the regulator *frmR*. J Bacteriol. 2004;186(20):6714-20.
28. Kasahara M, Nakata A, Shinagawa H. Molecular analysis of the *Escherichia coli phoP-phoQ* operon. J Bacteriol. 1992;174(2):492-8.
29. Ruiz N, Silhavy TJ. Sensing external stress: watchdogs of the *Escherichia coli* cell envelope. Curr Opin Microbiol. 2005;8(2):122-6.
30. Bontemps-Gallo S, Bohin J-P, Lacroix J-M, Slauch JM. Osmoregulated Periplasmic Glucans. EcoSal Plus. 2017;7(2).
31. Alexeeva S, Hellingwerf KJ, Teixeira de Mattos MJ. Requirement of ArcA for redox regulation in *Escherichia coli* under microaerobic but not anaerobic or aerobic conditions. J Bacteriol. 2003;185(1):204-9.
32. Hengge R, Galperin MY, Ghigo J-M, Gomelsky M, Green J, Hughes KT, et al. Systematic Nomenclature for GGDEF and EAL Domain-Containing Cyclic Di-GMP Turnover Proteins of *Escherichia coli*. J Bacteriol. 2015;198(1):7-11.
33. Roy A, Danchin A. The *cya* locus of *Escherichia coli* K12: Organization and gene products. Molecular and General Genetics MGG. 1982;188(3):465-71.
34. Chen R, Henning U. A periplasmic protein (Skp) of *Escherichia coli* selectively binds a class of outer membrane proteins. Mol Microbiol. 1996;19(6):1287-94.
35. Lazar SW, Kolter R. SurA assists the folding of *Escherichia coli* outer membrane proteins. J Bacteriol. 1996;178(6):1770-3.
36. Baars L, Ytterberg AJ, Drew D, Wagner S, Thilo C, van Wijk KJ, et al. Defining the role of the *Escherichia coli* chaperone SecB using comparative proteomics. J Biol Chem. 2006;281(15):10024-34.
37. Alba BM, Gross CA. Regulation of the *Escherichia coli* sigma-dependent envelope stress response. Mol Microbiol. 2004;52(3):613-9.
38. Szyf M, Avraham-Haetzni K, Reifman A, Shlomai J, Kaplan F, Oppenheim A, et al. DNA methylation pattern is determined by the intracellular level of the methylase. Proc Natl Acad Sci U S A. 1984;81(11):3278-82.
39. Valens M, Thiel A, Boccard F. The MaoP/*maoS* Site-Specific System Organizes the Ori Region of the *E. coli* Chromosome into a Macrodomain. PLoS Genet. 2016;12(9):e1006309-e.
40. Yarranton GT, Gefter ML. Enzyme-catalyzed DNA unwinding: studies on *Escherichia coli* rep protein. Proc Natl Acad Sci U S A. 1979;76(4):1658-62.
41. Schneider D, Kaiser W, Stutz C, Holinski A, Mayans O, Babinger P. YbiB from *Escherichia coli*, the Defining Member of the Novel TrpD2 Family of Prokaryotic DNA-binding Proteins. The Journal of biological chemistry. 2015;290(32):19527-39.
42. Yamaguchi Y, Inouye M. An endogenous protein inhibitor, YjhX (TopAI), for topoisomerase I from *Escherichia coli*. Nucleic Acids Res. 2015;43(21):10387-96.
43. Turner RJ, Taylor DE, Weiner JH. Expression of *Escherichia coli* TehA gives resistance to antiseptics and disinfectants similar to that conferred by multidrug resistance efflux pumps. Antimicrob Agents Chemother. 1997;41(2):440-4.
44. Lusetti SL, Drees JC, Stohl EA, Seifert HS, Cox MM. The DinI and RecX proteins are competing modulators of RecA function. J Biol Chem. 2004;279(53):55073-9.
45. Hickson ID, Robson CN, Atkinson KE, Hutton L, Emmerson PT. Reconstitution of RecBC DNase activity from purified *Escherichia coli* RecB and RecC proteins. J Biol Chem. 1985;260(2):1224-9.

46. Deacon J, Cooper RA. D-Galactonate utilisation by enteric bacteria. The catabolic pathway in *Escherichia coli*. FEBS Lett. 1977;77(2):201-5.
47. Brock M, Darley D, Textor S, Buckel W. 2-Methylisocitrate lyases from the bacterium *Escherichia coli* and the filamentous fungus *Aspergillus nidulans*: characterization and comparison of both enzymes. Eur J Biochem. 2001;268(12):3577-86.
48. Shafqat J, Höög JO, Hjelmqvist L, Oppermann UC, Ibáñez C, Jörnvall H. An ethanol-inducible MDR ethanol dehydrogenase/acetaldehyde reductase in *Escherichia coli*: structural and enzymatic relationships to the eukaryotic protein forms. Eur J Biochem. 1999;263(2):305-11.
49. Lee SJ, Ko JH, Kang HY, Lee Y. Coupled expression of MhpE aldolase and MhpF dehydrogenase in *Escherichia coli*. Biochem Biophys Res Commun. 2006;346(3):1009-15.
50. Hernandez-Montalvo V, Martinez A, Hernandez-Chavez G, Bolivar F, Valle F, Gosset G. Expression of *galP* and *glk* in a *Escherichia coli* PTS mutant restores glucose transport and increases glycolytic flux to fermentation products. Biotechnol Bioeng. 2003;83(6):687-94.
51. Lopilato JE, Garwin JL, Emr SD, Silhavy TJ, Beckwith JR. D-ribose metabolism in *Escherichia coli* K-12: genetics, regulation, and transport. J Bacteriol. 1984;158(2):665-73.
52. Hall RS, Xiang DF, Xu C, Raushel FM. N-Acetyl-D-glucosamine-6-phosphate deacetylase: substrate activation via a single divalent metal ion. Biochemistry. 2007;46(27):7942-52.
53. Penrod JT, Roth JR. Conserving a Volatile Metabolite: a Role for Carboxysome-Like Organelles in *Salmonella enterica*. J Bacteriol. 2006;188(8):2865-74.
54. Harborne NR, Griffiths L, Busby SJW, Cole JA. Transcriptional control, translation and function of the products of the five open reading frames of the *Escherichia coli nir* operon. Mol Microbiol. 1992;6(19):2805-13.
55. Schneider K, Dimroth P, Bott M. Identification of triphosphoribosyl-dephospho-CoA as precursor of the citrate lyase prosthetic group. FEBS Lett. 2000;483(2-3):165-8.
56. Weiner JH, Shaw G, Turner RJ, Trieber CA. The topology of the anchor subunit of dimethyl sulfoxide reductase of *Escherichia coli*. J Biol Chem. 1993;268(5):3238-44.
57. Fuchs JA, Warner HR. Isolation of an *Escherichia coli* mutant deficient in glutathione synthesis. J Bacteriol. 1975;124(1):140-8.
58. Niehaus TD, Elbadawi-Sidhu M, de Crécy-Lagard V, Fiehn O, Hanson AD. Discovery of a widespread prokaryotic 5-oxoprolinase that was hiding in plain sight. J Biol Chem. 2017;292(39):16360-7.
59. Thèze J, Margarita D, Cohen GN, Borne F, Patte JC. Mapping of the structural genes of the three aspartokinases and of the two homoserine dehydrogenases of *Escherichia coli* K-12. J Bacteriol. 1974;117(1):133-43.
60. Gemmill RM, Jones JW, Haughn GW, Calvo JM. Transcription initiation sites of the leucine operons of *Salmonella* typhimurium and *Escherichia coli*. J Mol Biol. 1983;170(1):39-59.
61. Van Vliet F, Crabeel M, Boyen A, Tricot C, Stalon V, Falmagne P, et al. Sequences of the genes encoding argininosuccinate synthetase in *Escherichia coli* and *Saccharomyces cerevisiae*: comparison with methanogenic archaebacteria and mammals. Gene. 1990;95(1):99-104.
62. Suzuki H, Nishimura Y, Hirota Y. On the process of cellular division in *Escherichia coli*: a series of mutants of *E. coli* altered in the penicillin-binding proteins. Proc Natl Acad Sci U S A. 1978;75(2):664-8.
63. Typas A, Banzhaf M, van den Berg van Saparoea B, Verheul J, Biboy J, Nichols RJ, et al. Regulation of peptidoglycan synthesis by outer-membrane proteins. Cell. 2010;143(7):1097-109.
64. Zawadzke LE, Bugg TD, Walsh CT. Existence of two D-alanine:D-alanine ligases in *Escherichia coli*: cloning and sequencing of the *ddlA* gene and purification and characterization of the DdlA and DdlB enzymes. Biochemistry. 1991;30(6):1673-82.
65. Hara H, Yamamoto Y, Higashitani A, Suzuki H, Nishimura Y. Cloning, mapping, and characterization of the *Escherichia coli* prc gene, which is involved in C-terminal processing of penicillin-binding protein 3. J Bacteriol. 1991;173(15):4799-813.
66. Templin MF, Ursinus A, Höltje JV. A defect in cell wall recycling triggers autolysis during the stationary growth phase of *Escherichia coli*. EMBO J. 1999;18(15):4108-17.
67. Tsang MJ, Yakhnina AA, Bernhardt TG. NlpD links cell wall remodeling and outer membrane invagination during cytokinesis in *Escherichia coli*. PLoS Genet. 2017;13(7):e1006888.
68. Fath MJ, Mahanty HK, Kolter R. Characterization of a *purF* operon mutation which affects colicin V production. J Bacteriol. 1989;171(6):3158-61.

69. Gerding MA, Ogata Y, Pecora ND, Niki H, de Boer PA. The trans-envelope Tol-Pal complex is part of the cell division machinery and required for proper outer-membrane invagination during cell constriction in *E. coli*. Mol Microbiol. 2007;63(4):1008-25.
70. Meier-Dieter U, Starman R, Barr K, Mayer H, Rick PD. Biosynthesis of enterobacterial common antigen in *Escherichia coli.* Biochemical characterization of Tn10 insertion mutants defective in enterobacterial common antigen synthesis. J Biol Chem. 1990;265(23):13490-7.
71. Islam ST, Lam JS. Synthesis of bacterial polysaccharides via the Wzx/Wzy-dependent pathway. Can J Microbiol. 2014;60(11):697-716.
72. Mitchell AM, Srikumar T, Silhavy TJ, Hultgren SJ. Cyclic Enterobacterial Common Antigen Maintains the Outer Membrane Permeability Barrier of *Escherichia coli* in a Manner Controlled by YhdP. mBio. 2018;9(4):e01321-18.
73. Franco AV, Liu D, Reeves PR. A Wzz (Cld) protein determines the chain length of K lipopolysaccharide in *Escherichia coli* O8 and O9 strains. J Bacteriol. 1996;178(7):1903-7.
74. Ma X, Prathapam R, Wartchow C, Chie-Leon B, Ho CM, De Vicente J, et al. Structural and Biological Basis of Small Molecule Inhibition of *Escherichia coli* LpxD Acyltransferase Essential for Lipopolysaccharide Biosynthesis. ACS infectious diseases. 2020;6(6):1480-9.
75. Wang Z, Wang J, Ren G, Li Y, Wang X. Influence of Core Oligosaccharide of Lipopolysaccharide to Outer Membrane Behavior of *Escherichia coli*. Mar Drugs. 2015;13(6):3325-39.
76. Wang X, Quinn PJ. Lipopolysaccharide: Biosynthetic pathway and structure modification. Prog Lipid Res. 2010;49(2):97-107.
77. Tomar SK, Kumar P, Prakash B. Deciphering the catalytic machinery in a universally conserved ribosome binding ATPase YchF. Biochem Biophys Res Commun. 2011;408(3):459-64.
78. Kang WK, Icho T, Isono S, Kitakawa M, Isono K. Characterization of the gene *rimK* responsible for the addition of glutamic acid residues to the C-terminus of ribosomal protein S6 in *Escherichia coli* K12. Mol Gen Genet. 1989;217(2-3):281-8.
79. Yoshida H, Maki Y, Furuike S, Sakai A, Ueta M, Wada A. YqjD is an inner membrane protein associated with stationary-phase ribosomes in *Escherichia coli*. J Bacteriol. 2012;194(16):4178-83.
80. Sakai Y, Kimura S, Suzuki T. Dual pathways of tRNA hydroxylation ensure efficient translation by expanding decoding capability. Nature communications. 2019;10(1):2858-.
81. Caserta E, Tomšic J, Spurio R, La Teana A, Pon CL, Gualerzi CO. Translation Initiation Factor IF2 Interacts with the 30 S Ribosomal Subunit via Two Separate Binding Sites. J Mol Biol. 2006;362(4):787-99.
82. Weijland A, Harmark K, Cool RH, Anborgh PH, Parmeggiani A. Elongation factor Tu: a molecular switch in protein biosynthesis. Mol Microbiol. 1992;6(6):683-8.
83. Peil L, Virumäe K, Remme J. Ribosome assembly in *Escherichia coli* strains lacking the RNA helicase DeaD/CsdA or DbpA. FEBS J. 2008;275(15):3772-82.
84. Resch A, Većerek B, Palavra K, Bläsi U. Requirement of the CsdA DEAD-box helicase for low temperature riboregulation of *rpoS* mRNA. RNA Biol. 2010;7(6):796-802.
85. Choi E, Hwang J. The GTPase BipA expressed at low temperature in *Escherichia coli* assists ribosome assembly and has chaperone-like activity. J Biol Chem. 2018;293(47):18404-19.
86. Sheidy DT, Zielke RA. Analysis and expansion of the role of the *Escherichia coli* protein ProQ. PLoS One. 2013;8(10):e79656.
87. Klemm P. Two regulatory *fim* genes, *fimB* and *fimE*, control the phase variation of type 1 fimbriae in *Escherichia coli*. The EMBO Journal. 1986;5(6):1389-93.
88. Saini S, Pearl JA, Rao CV. Role of FimW, FimY, and FimZ in Regulating the Expression of Type I Fimbriae in *Salmonella enterica* Serovar Typhimurium. J Bacteriol. 2009;191(9):3003-10.
89. Allen WJ, Phan G, Waksman G. Pilus biogenesis at the outer membrane of Gram-negative bacterial pathogens. Curr Opin Struct Biol. 2012;22(4):500-6.
90. Cisneros DA, Pehau-Arnaudet G, Francetic O. Heterologous assembly of type IV pili by a type II secretion system reveals the role of minor pilins in assembly initiation. Mol Microbiol. 2012;86(4):805-18.
91. Lehnen D, Blumer C, Polen T, Wackwitz B, Wendisch VF, Unden G. LrhA as a new transcriptional key regulator of flagella, motility and chemotaxis genes in *Escherichia coli*. Mol Microbiol. 2002;45(2):521-32.

92. Ko M, Park C. H-NS-Dependent regulation of flagellar synthesis is mediated by a LysR family protein. J Bacteriol. 2000;182(16):4670-2.
93. Kasimoglu E, Park SJ, Malek J, Tseng CP, Gunsalus RP. Transcriptional regulation of the proton-translocating ATPase (*atpIBEFHAGDC*) operon of *Escherichia coli*: control by cell growth rate. J Bacteriol. 1996;178(19):5563-7.
94. Maciag A, Peano C, Pietrelli A, Egli T, De Bellis G, Landini P. *In vitro* transcription profiling of the σS subunit of bacterial RNA polymerase: re-definition of the σS regulon and identification of σS-specific promoter sequence elements. Nucleic Acids Res. 2011;39(13):5338-55.
95. Figueroa-Bossi N, Uzzau S, Maloriol D, Bossi L. Variable assortment of prophages provides a transferable repertoire of pathogenic determinants in *Salmonella*. Mol Microbiol. 2001;39(2):260-71.
96. Monteiro DCF, Patel V, Bartlett CP, Nozaki S, Grant TD, Gowdy JA, et al. The structure of the PanD/PanZ protein complex reveals negative feedback regulation of pantothenate biosynthesis by coenzyme A. Chem Biol. 2015;22(4):492-503.
97. Santander PJ, Kajiwara Y, Williams HJ, Scott AI. Structural characterization of novel cobalt corrinoids synthesized by enzymes of the vitamin B12 anaerobic pathway. Bioorg Med Chem. 2006;14(3):724-31.
98. Marteyn BS, Karimova G, Fenton AK, Gazi AD, West N, Touqui L, et al. ZapE is a novel cell division protein interacting with FtsZ and modulating the Z-ring dynamics. mBio. 2014;5(2):e00022-14.
